# Cryo-balloon catheter localization in X-Ray fluoroscopy using U-net

**DOI:** 10.1007/s11548-021-02366-5

**Published:** 2021-04-20

**Authors:** Ina Vernikouskaya, Dagmar Bertsche, Tillman Dahme, Volker Rasche

**Affiliations:** Department of Internal Medicine II, Ulm University Medical Center, Albert-Einstein-Allee 23, 89081 Ulm, Germany

**Keywords:** Cryo-balloon, Automatic segmentation, Reconstruction, Semi-automatic annotation, Unet

## Abstract

**Purpose:**

Automatic identification of interventional devices in X-ray (XR) fluoroscopy offers the potential of improved navigation during transcatheter endovascular procedures. This paper presents a prototype implementation of fully automatic 3D reconstruction of a cryo-balloon catheter during pulmonary vein isolation (PVI) procedures by deep learning approaches.

**Methods:**

We employ convolutional neural networks (CNN) to automatically identify the cryo-balloon XR marker and catheter shaft in 2D fluoroscopy during PVI. Training data are generated exploiting established semiautomatic techniques, including template-matching and analytical graph building. A first network of U-net architecture uses a single grayscale XR image as input and yields the mask of the XR marker. A second network of the similar architecture is trained using the mask of the XR marker as additional input to the grayscale XR image for the segmentation of the cryo-balloon catheter shaft mask. The structures automatically identified in two 2D images with different angulations are then used to reconstruct the cryo-balloon in 3D.

**Results:**

Automatic identification of the XR marker was successful in 78% of test cases and in 100% for the catheter shaft. Training of the model for prediction of the XR marker mask was successful with 3426 training samples. Incorporation of the XR marker mask as additional input for the model predicting the catheter shaft allowed to achieve good training result with only 805 training samples. The average prediction time per frame was 14.47 ms for the XR marker and 78.22 ms for the catheter shaft. Localization accuracy for the XR marker yielded on average 1.52 pixels or 0.56 mm.

**Conclusions:**

In this paper, we report a novel method for automatic detection and 3D reconstruction of the cryo-balloon catheter shaft and marker from 2D fluoroscopic images. Initial evaluation yields promising results thus indicating the high potential of CNNs as alternatives to the current state-of-the-art solutions.

## Introduction

Atrial fibrillation is a common heart arrhythmia associated with an increased risk of stroke. The current state-of-the-art treatment option is minimally invasive catheter ablation. Cryo-balloon catheters isolate a pulmonary vein by freezing the tissue annularly around its ostium using liquid nitrogen injected into the balloon device. The planning of cryo-balloon ablations of atrial fibrillation is a crucial task for a physician as determining the correct size of the balloon catheter is required for successful isolation of each pulmonary vein (PV). The radio-opaque marker of the cryo-balloon localized in the biplane X-ray (XR) fluoroscopy facilitates the verification of the balloon catheter position with respect to the preoperative data set of the left atrium [1]. Further intra-procedural support can be provided by visualizing the position and dimensions of the cryo-balloon catheter overlaid onto the fluoroscopic images using overlay guidance systems (OGS) [2, 3]. Based on the knowledge of its 3D XR marker position, vector of the catheter shaft, and diameter, several methods for the reconstruction of a 3D model of the cryo-balloon have been introduced with reported submillimeter levels of accuracy for sphere reconstruction [1, 4, 5]. Also, methods to reconstruct ellipsoids requiring three views [6] or spheroids from even a single image plane [7] have been reported. The previously addressed 3D reconstruction methods will significantly benefit from automatic detection of the radio-opaque marker and the catheter shaft in the fluoroscopic images, as it has been shown for automatic 3D catheter tracking inside the patient’s vascular tree [8] or in electrophysiology (EP) procedures [9, 10]. Therefore, the purpose of this work is to fully automatically identify the cryo-balloon marker and the catheter shaft in 2D XR fluoroscopy to be further used for initialization of the 3D reconstruction method of the cryo-balloon, exemplarily addressed in this paper.

Although convolutional neural networks (CNN) are extensively used for catheter detection and tracking, a large amount of annotated training data is required for all deep learning approaches for pixel-level segmentation. Manual pixel-level labeling is laborious and time-consuming; thus, methods reducing the required manual interaction will greatly facilitate the development of CNN models for semantic segmentation [11].

In this work, we propose to use networks of similar encoder-decoder U-net architecture to detect separately the XR marker and the cryo-balloon catheter shaft in the XR images from cryo-balloon pulmonary vein isolation (PVI) procedures. For training of the networks, semiautomatically annotated training data were used. The annotated training data for marker prediction were used additionally to the catheter shaft training data for further improvement of prediction capability and simultaneous reduction of the required amount of training data.

## Methods

### CNN model

**Fig. 1:**
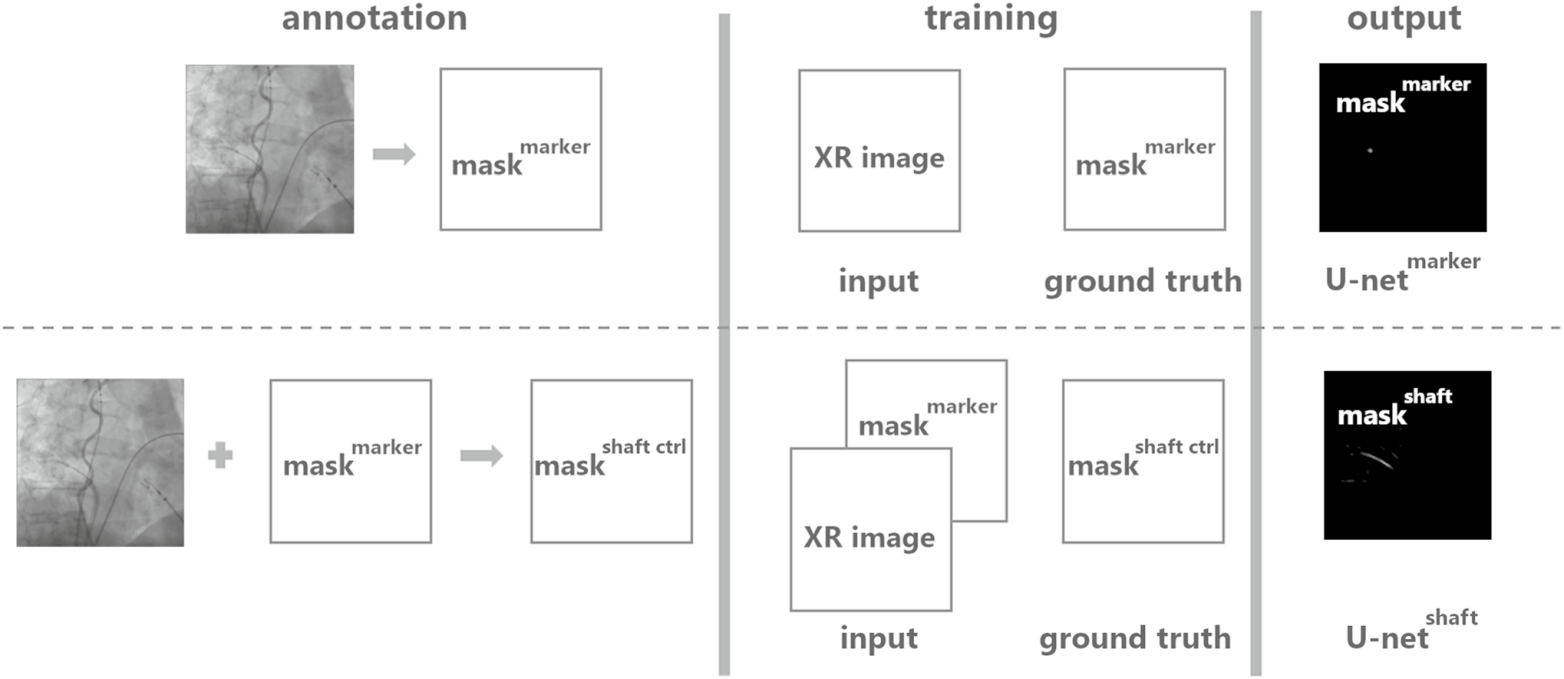
Method processing algorithm subdivided into annotation, training, and output steps. Annotation step: the mask of the cryo-balloon marker mask^marker^ is obtained from the original XR image to be processed by the U-net^marker^; the mask of the cryo-balloon catheter shaft centerline mask^shaft ctrl^ is obtained from the original XR image and its corresponding mask^marker^ to be processed by the U-net^shaft^. Training step: U-net^marker^ is trained on the image sample consisting of original XR image and its corresponding mask^marker^ as ground truth; U-net^shaft^ is trained on the image sample consisting of original XR image and its corresponding mask^marker^ as inputs and mask^shaft ctrl^ as ground truth. Output step: mask^marker^ and mask^shaft^ are predicted by the U-net^marker^ and U-net^shaft^ correspondingly as images containing contours of specific pixel values against background

The proposed method utilizes an adapted CNN model of the well-known U-net architecture to segment the radio-opaque marker (U-net^marker^) and the cryo-balloon catheter shaft (U-net^shaft^) separately in the XR images from PVI procedures. The adapted U-net model consists of a convolutional part downsampled with the maxpool layer and strided transposed convolutional upsampling part in combination with dropout regularization and residual connection to the output and was previously described by our group [12]. Both models were implemented in Keras and trained on a GeForce GTX 1060 6 GB GPU for 50 epochs with a batch size of 8 (U-net^marker^) or 4 (U-net^shaft^) samples per pass with the adaptive moment estimation algorithm. U-net^marker^ uses a lossless grayscale XR image as input and predicts the radio-opaque marker as a mask (mask^marker^). U-net^shaft^ consists of two inputs: current lossless grayscale XR image and mask^marker^, and predicts the catheter shaft mask (mask^shaft^). The implemented algorithm showing the annotation and training steps with corresponding inputs/outputs, as well as predicted outputs of both models is sketched in Fig. 1.

All available XR data were pre-processed and annotated semiautomatically as described below in Annotation.

### Data

Eighty-nine fluoroscopic runs from 15 different PVI patients acquired on biplane C-arm X-ray system (Allura Xper, Philips Healthcare, Best, The Netherlands) were used for development and evaluation of the proposed method. Two patients’ data were acquired in monoplane mode (44 runs in total), 13 patients’ data—in biplane mode (45 runs in total, on average 2 frontal and 2 lateral runs per patient). A total of 3426 XR images of 512 × 512 pixels resolution (all 76 available runs from 11 patients) were successfully annotated and used for training/validation of U-net^marker^. A total of 805 images (32 biplane runs from 10 patients and 2 monoplane runs from 1 patient) were used to train/validate the U-net^shaft^. A total of 508 XR images (13 biplane runs from 4 patients) were used for testing both models.

### Annotation

**Fig. 2:**
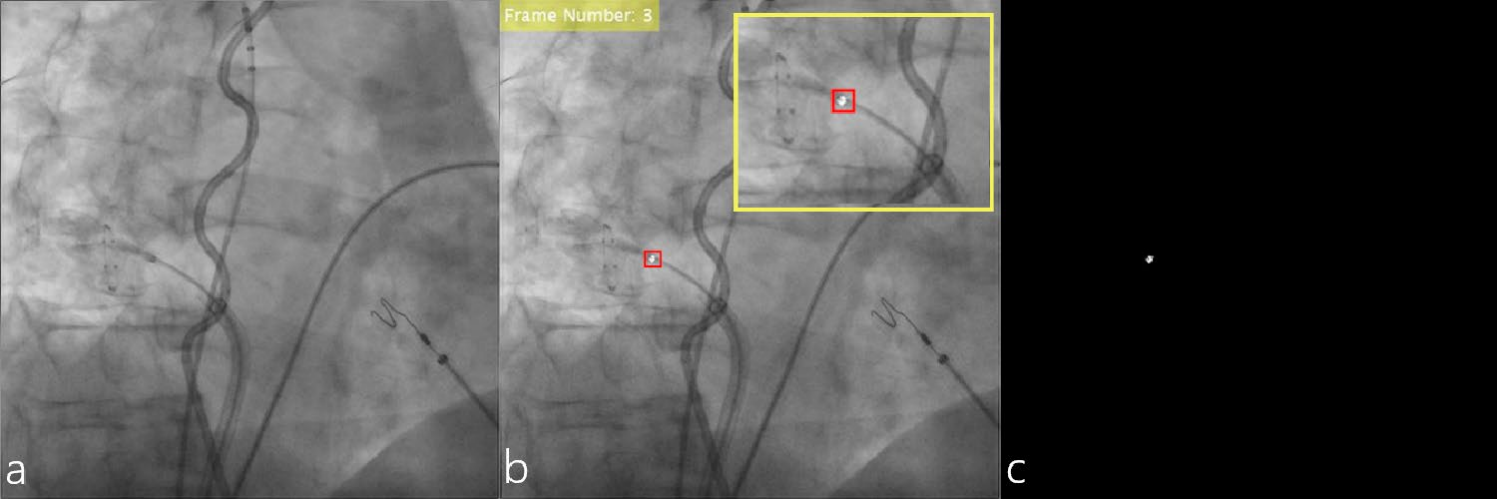
Semiautomatic annotation of the cryo-balloon marker: **a** original XR image; **b** XR frame with a rectangular template of 10 × 10 pixels set around the XR marker (red box) and overlaid thresholded pixels corresponding to the segmented marker (incl. close-up of the respective region of interest); **c** resulting binary mask mask^marker^

The radio-opaque marker mask (mask^marker^) was created by thresholding the signal intensity values of the XR image (Fig. 2a) within a 10 × 10 pixels region of interest (ROI) around the marker (Fig. 2b). A threshold value for marker detection was identified individually for each run based on the intensity values of the marker in the first frame. A single point within the marker was manually set in the first frame of each run and extended to the rectangular shaped template. ROI position through the run was automatically adjusted applying template-matching using the local sums to normalize the cross-correlation in MATLAB. Finally, a series of binary masks of the XR marker corresponding to each frame in the XR run was created (Fig. 2c). For each frame in the XR run, the created binary mask was overlaid on the XR image and reviewed visually for accuracy (Fig. 2b, close-up); the outliers were removed.

**Fig. 3:**
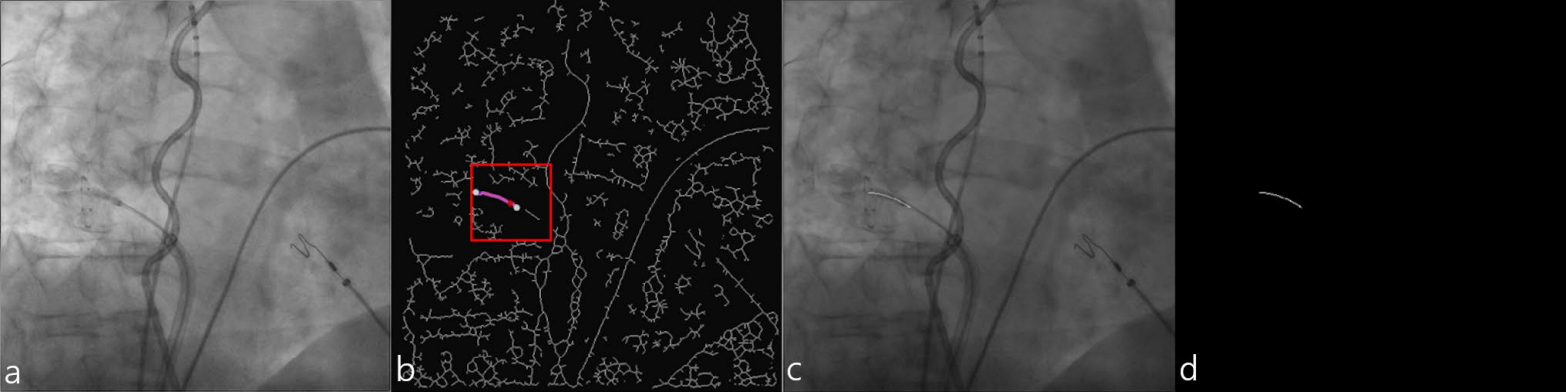
Semiautomatic annotation of the cryo-balloon catheter shaft: **a** original XR image; **b** skeletonized binary image with the analytical graph (magenta) built within the 50 × 50 pixels ROI (red rectangular box) around the seed point (red point) and two end nodes (white points) indicating the longest path closest to the seed point and interpreted as a catheter shaft; **c** XR frame with overlaid resulting spline fitted longest path providing the centerline of the catheter shaft; **d** resulting binary mask mask^shaft ctrl^

Catheter shaft binary mask (mask^shaft ctrl^) was created from a centerline model build similarly as described in [4, 13] as the analytical representation of the cryo-balloon catheter shaft part close to the XR marker. First, the following image pre-processing steps were undertaken: each 8-bit integer grayscale XR image (Fig. 3a) was inverted, histogram equalized, enhanced applying a hybrid Hessian-based filter [14], binarized, and skeletonized. The resulting image can be seen in Fig. 3b.

After pre-processing, the search area for the catheter shaft in the skeletonized image was reduced to 50 × 50 pixels ROI around the marker (Fig. 3b, red rectangular box). The ROI center is given by the center of the minimum-area bounding rectangle of the contour provided by the mask^marker^ and will be further considered as a seed point (Fig. 3b, red point). To close small gaps, a closing operation was performed before skeletonization. The skeleton within the search area was then transformed into a single gapless graph (Fig. 3b, magenta points) initialized by the seed point. The shortest paths between all end nodes of the graph were computed by Dijkstra’s algorithm. From the resulting paths, the longest path closest to the seed point located within an initially predefined catheter orientation dependent interval was considered as estimate for the catheter shaft (Fig. 3b, all nodes between two white points). The resulting centerline was then obtained by spline fitting of the computed path and overlaid onto the XR image for visual reviewing of the segmentation accuracy (Fig. 3c). In case of failure, the procedure was redone with another pre-defined interval. An exemplarily resulting binary mask mask^shaft ctrl^ generated for the training of U-net^shaft^ is shown in Fig. 3d. The complete algorithm was implemented in Python using OpenCV and SciPy libraries for image processing and NetworkX library for building the graphs.

### Post-processing and evaluation

Generated outputs of both models—mask^marker^ and mask^shaft^—are images with detected contours given by the specific nonzero pixel values on the black background. To obtain a single seed point corresponding to XR marker from predicted mask^marker^ thresholded at 20% of maximum intensity, the centroid of the detected contour was calculated using image moments in OpenCV. For accuracy evaluation, the average Euclidean distance between the calculated seed point and centroid of the annotated marker contour was calculated. To compute the catheter shaft centerline, the mask^shaft^ was thresholded at 1.5% of maximum intensity and skeletonized. Resulting centerline coordinates were obtained by spline fitting of the longest path computed for a graph built for the contour initialized by the computed seed point.

### 3D reconstruction

**Fig. 4.**
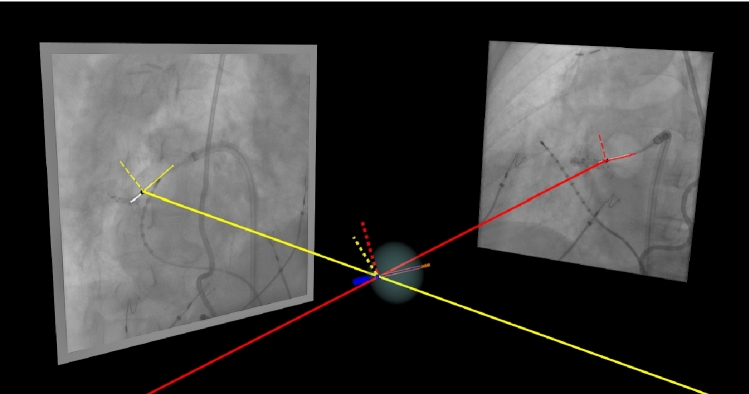
3D reconstruction of the automatically detected seed points (black points in both XR images) and catheter shaft centerlines (white lines in both XR images) from frontal view acquired in RAO30° orientation (red lines) and lateral view acquired in LAO40° orientation (yellow lines) and alignment with the previously constructed 28 mm-sized cryo-balloon model. Solid red and yellow lines represent projection vectors for frontal and lateral views, respectively. Light red and yellow lines on both XR images represent respective line fitted centerlines. Dashed red and yellow lines on both XR images represent respective orthonormals, whereas dotted red and yellow lines represent transferred orthonormals initialized by the reconstructed 3D marker position aligned with the cryo-balloon model marker (dark blue small ellipsoid). Solid orange line represents resulting catheter orientation vector aligned with the cryo-balloon model shaft (dark blue line)

The resulting seed point and spline fitted centerline for each XR image were used for 3D reconstruction of the cryo-balloon marker and catheter shaft from two views. The reconstructed 3D marker position and catheter shaft direction vector were aligned with the previously constructed 3D model of the 28 mm-sized cryo-balloon designed according to Arctic Front Advance Pro™ specifications (Medtronic, Minneapolis, USA), including the balloon ellipsoid, catheter shaft with tip, and radio-opaque marker (Fig. 4). With the known geometry of the XR system for each specific view, the 3D position of the radio-opaque marker given by the seed point in each view (Fig. 4, black points) was reconstructed based on epipolar geometry [15]. Created centerlines in both biplane images (Fig. 4, white lines) were line fitted (Fig. 4, red light vector on frontal view and yellow light vector on lateral view) using minimal averaged Euclidean distance to obtain the associated unit direction vector. Applying the cross-product on the fitted lines and the corresponding projection vector (Fig. 4, red and yellow solid lines correspondingly), orthonormals (Fig. 4, red and yellow dashed lines) were obtained. The catheter orientation (Fig. 4, orange) resulted from the cross-product of both orthonormals, transferred according to projection geometry to the previously determined 3D marker position (Fig. 4, red and yellow dotted lines).

Since no 3D data were available as ground truth, the result of 3D reconstruction was back-projected into the image planes and the deviation of the re-projected XR marker and direction vector was calculated using the Euclidean distance between the seed points and re-projected reconstructed XR markers.

## Results

The XR marker of the cryo-balloon could be visually detected in 394 (78% of cases) test samples, whereas 3426 image samples were used for training and validation. A total of 805 training/validation samples were required to achieve 100% visual detectability of the catheter shaft (Table 1). As expected, the accuracy of the method is limited by the presence of interfering features, e.g., injected contrast agent overlaying the target structures, other catheters, vessel-like structures, and electrodes, in the image field of view.

**Table 1:** Summary of the results from test runs provided by U-net^marker^ and U-net^shaft^

XR test run number	C-arm	Projection orientation	Number of frames in XR run	U-net^marker^			U-net^shaft^	
				Number of frames with detected marker	Prediction time per frame (ms)	Average Euclidean distance (pxl)	Number of frames with detected catheter shaft	Prediction time per frame (ms)
1	frontal	RAO30°	58	58	11.58	1.22	58	58.73
2	frontal	RAO30°	49	0	12.12	–	49	63.78
3	lateral	LAO40°	19	0	14.80	–	19	93.75
4	lateral	LAO40°	54	54	11.86	1.12	54	60.76
5	frontal	RAO30°	33	33	13.73	1.15	33	77.18
6	frontal	RAO30°	19	19	15.63	2.77	19	94.57
7	lateral	LAO40°	36	36	13.02	1.38	36	74.22
8	lateral	LAO40°	10	10	28.13	1.5	10	148.44
9	frontal	RAO30°	41	17	12.58	2.6	41	70.88
10	frontal	RAO30°	87	87	11.85	1.19	87	44.72
11	lateral	LAO40°	42	42	12.28	0.78	42	66.59
12	frontal	RAO30°	38	38	15.63	1.5	38	72.37
13	lateral	LAO40°	22	0	14.91	–	22	90.91
Average					14.47	1.52		78.22

Even if the marker could not be detected automatically with U-net^marker^ (Table 1, Test runs 2, 3, 9, and 13), incorporating the annotated mask^marker^ as additional input for U-net^shaft^ yielded accurate detection of the part of the cryo-balloon catheter shaft close to the seed point.

The average time to predict the whole mask of 512 × 512 pixels resolution was 14.47 ms for the XR marker and 78.22 ms for the catheter shaft.

**Fig. 5:**
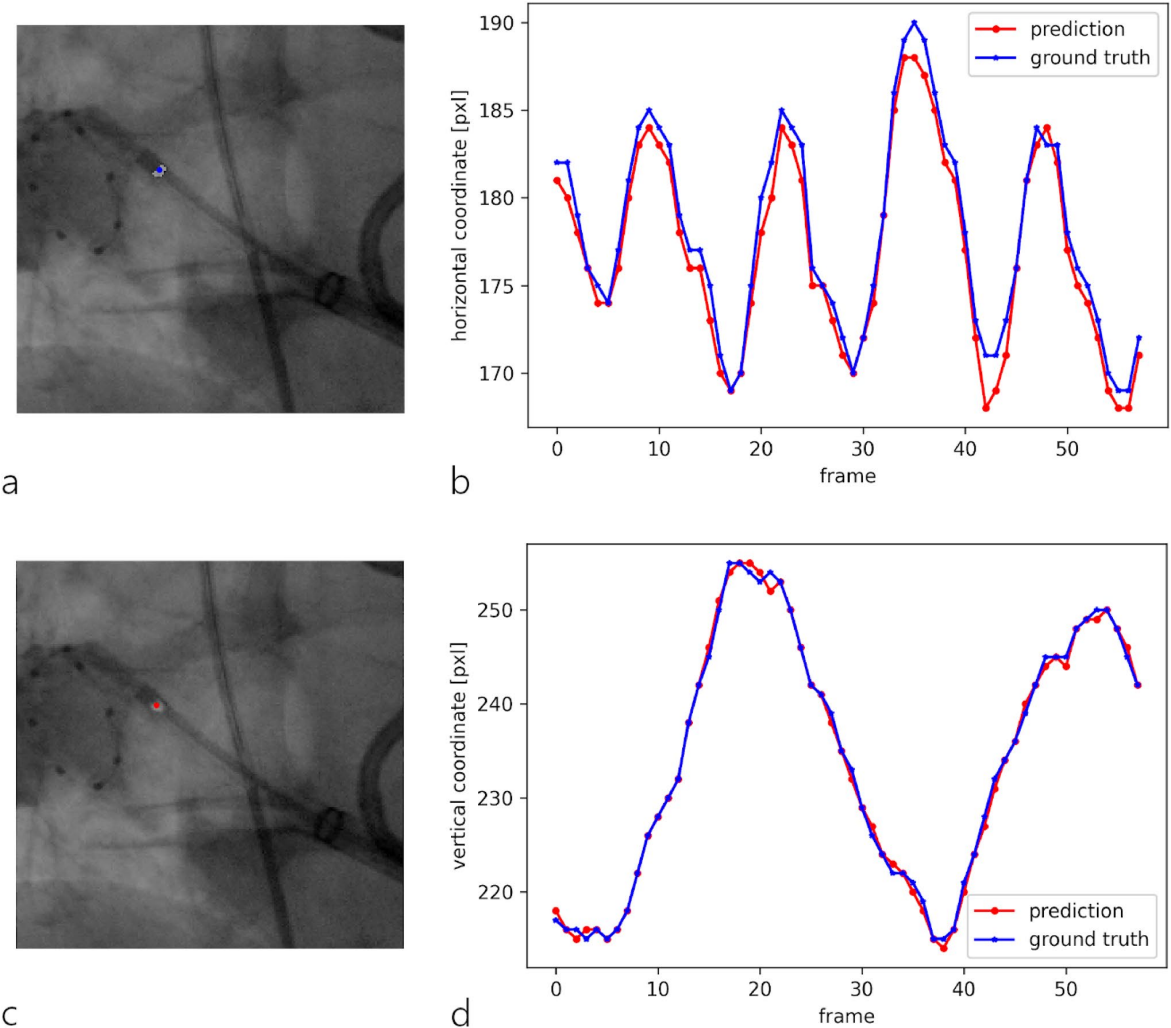
Evaluation of the XR marker prediction accuracy from test run 1: cutout of the annotated mask^marker^ (**a**) or predicted mask^marker^ (**c**) overlaid on original XR image with the colored point indicating the centroid of the annotated marker contour as ground truth (blue) or the centroid of the predicted contour (red); plot of the predicted centroid’s horizontal (**b**) and vertical (**d**) coordinates overlaid on the ground truth coordinates

Despite an average deviation of 1.52 pixels or 0.56 mm, an accurate overlap between the centroids of the contours provided by the predicted XR marker mask and the ground truth mask could be achieved for all test data sets where the XR marker was detected (Fig. 5).

**Fig. 6:**
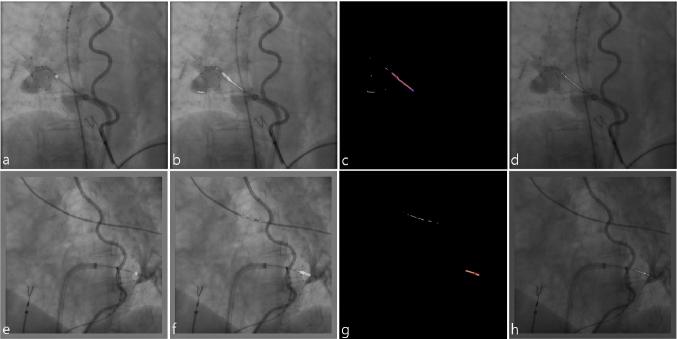
Exemplary results of test run 1 (**a–d**) and test run 4 (**e–h**): **a**, **e** output of U-net^marker^ overlaid on original XR image; **b**, **f** output of U-net^shaft^ overlaid on original XR image; **c**, **g** the post-processed binary image representing the skeleton of the image outputted by U-net^shaft^ with the overlaid graph and a center of the contour detected by U-net^marker^ (red point); **d**, **h** resulting seed point and spline fitted centerline overlaid on original XR image

In direct comparison with the image processing algorithms utilized for the creation of the annotated training data, where the Hessian filter was often not able to manage the appearance of gaps in the near vicinity of the seed point, the detection of the catheter shaft with U-net was flawless (Fig. 6).

**Fig. 7:**
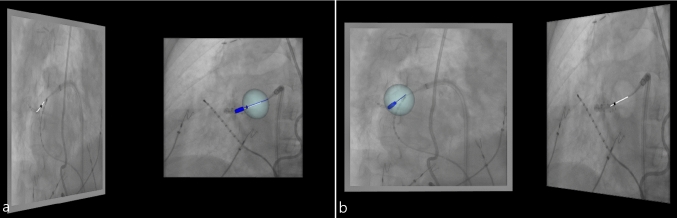
Projection of the constructed 28 mm-sized cryo-balloon model with ellipsoidal balloon (light blue) and catheter shaft (dark blue) with the tip (dark blue cylinder) and XR marker (dark blue small ellipsoid) onto RAO30° view acquired on the frontal C-arm (**a**) and LAO40° view acquired on the lateral C-arm (**b**). Automatically detected seed points are shown as black points in both XR images; catheter shaft centerlines are shown as white lines in both XR images

Figure 7 demonstrates an example of projection of the constructed cryo-balloon model aligned to the 3D structures (XR marker and direction vector) reconstructed from the automatically detected seed points and catheter centerlines in two views acquired in RAO30° on the frontal C-arm (Fig. 7a) and LAO40° on the lateral C-arm (Fig. 7b) onto these views. The aligned 28 mm-sized cryo-balloon model fits the corresponding marker and catheter shaft in the XR images with the deviation of back-projected marker of 0.33 mm or 0.9 pixels in frontal and 0.28 mm or 0.77 pixels in lateral view in this concrete example.

## Discussion and conclusions

The introduced method yields the possibility of automatic detection of the cryo-balloon catheter shaft and its marker in 2D fluoroscopic images using convolutional neural networks for localization of the cryo-balloon in 3D. The proposed annotation methods enable an automatic generation of the training data. However, for ensuring annotation fidelity, manual reviewing of the accuracy is required. Especially in the case of centerline generation, the review implicates time-consuming manual effort. This effort could be, however, reduced by incorporating the mask^marker^ as additional input for the U-net^shaft^ and thus generation of less annotated data required for successful end-to-end training of U-net^shaft^.

The method is still facing some limitations in the detection of XR marker and catheter shaft in case of injected contrast agent overlaying the target structures and difficult catheter orientations (e.g., highly angulated catheters). This is most likely due to the very limited availability of similar training data in which proper annotation was neither successful with the automatic approach nor manually. Moreover, the approach to uniquely identify the respective XR marker and catheter shaft in case of multiple detected regions in the predicted masks should be developed.

3D reconstruction of the cryo-balloon was successful by combining the 3D reconstruction of the XR marker using epipolar geometry with the reconstruction of the 3D directional vector of the catheter shaft. For successful 3D reconstruction, detection of the XR marker and catheter shaft in both views should be guaranteed by the neural networks. The accuracy of the reconstruction method is limited by the calibration accuracy of the biplane XR system and is restricted to no or very low catheter shaft angulation, as only a direction vector fitted to the centerline spline is considered for reconstruction.
